# Inhibition of COX2/PGD2-Related Autophagy Is Involved in the Mechanism of Brain Injury in T2DM Rat

**DOI:** 10.3389/fncel.2019.00068

**Published:** 2019-02-27

**Authors:** Yang Yang, Qi Chen, Quanfeng Zhao, Ying Luo, Ying Xu, Weimin Du, Hong Wang, Huan Li, Lu Yang, Congli Hu, Jiahua Zhang, Yuke Li, Hui Xia, Zhihao Chen, Jie Ma, Xiaoyan Tian, Junqing Yang

**Affiliations:** ^1^Department of Pharmacology, Chongqing Medical University, The Key Laboratory of Biochemistry and Molecular Pharmacology, Chongqing, China; ^2^Department of Pharmacy, GuiZhou Provincial People’s Hospital, Guiyang, China; ^3^Department of Pharmacy, Southwest Hospital, First Affiliated Hospital to TMMU, Third Military Medical University (Army Medical University), Chongqing, China; ^4^Department of Pharmaceutical Sciences, School of Pharmacy and Pharmaceutical Sciences, University at Buffalo, The State University of New York (SUNY), Buffalo, NY, United States

**Keywords:** cyclooxygenase-2, PGD2, type 2 diabetes, brain injury, autophagy

## Abstract

The present study was designed to observe the effect of COX2/PGD2-related autophagy on brain injury in type 2 diabetes rats. The histopathology was detected by haematoxylin–eosin staining. The learning and memory functions were evaluated by Morris water maze. The levels of insulin and PGD2 were measured by enzyme-linked immunosorbent assay. The expressions of COX2, p-AKT(S473), p-AMPK(T172), Aβ, Beclin1, LC3BII, and p62 were measured by immunohistochemistry and Western blotting. In model rats, we found that the body weight was significantly decreased, the blood glucose levels were significantly increased, the plasma insulin content was significantly decreased, the learning and memory functions were impaired and the cortex and hippocampus neurons showed significant nuclear pyknosis. The levels of COX2, p-AKT(S473), PGD2, Aβ, Beclin1 and p62 were significantly increased, whereas the expression of p-AMPK(T172) and LC3BII was significantly decreased in the cortex and hippocampus of model rats. In meloxicam-treated rats, the body weight, blood glucose and the content of plasma insulin did not significantly change, the learning and memory functions were improved and nuclear pyknosis was improved in the cortex and hippocampus neurons. The expression of p-AMPK(T172), Beclin1 and LC3BII was significantly increased, and the levels of COX2, p-AKT(S473), PGD2, Aβ, and p62 were significantly decreased in the cortex and hippocampus of meloxicam-treated rats. Our results suggested that the inhibition of COX2/PGD2-related autophagy was involved in the mechanism of brain injury caused by type 2 diabetes in rats.

## Introduction

The prevalence of type 2 diabetes mellitus (T2DM) is increasing with increased population aging and lifestyle changes. T2DM has become the main reason of disability and death in recent years (Sima, 2010). Diabetes can induce multiple organ damage. The central nervous system damage caused by diabetes has always received attention. A long-term study reported that the cognitive function declines at an accelerated pace in T2DM patients (Strachan et al., 2003; Wu et al., 2003; Hassing et al., 2004). In the mini-mental state examination test, the T2DM patients’ scores were reportedly lower than those of age-, sex- and educational level-matched non-diabetic controls (Ryan and Geckle, 2000; Xu et al., 2004). Studies have shown that the prevalence of cognitive dysfunction caused by diabetes is 25 to 36% (Alagiakrishnan et al., 2013). A major manifestation of brain injury caused by diabetes was attention deficiency, slower information processing, impaired learning ability, and memory decline. Although various factors are reportedly involved in T2DM-induced brain injury, the mechanism of damage is still unclear.

Cyclooxygenase (COX), a rate-limiting enzyme in the synthesis of prostaglandins (PGs), is involved in regulating various functions of the central nervous system (Yagami et al., 2016). COX includes the structural COX1 and the inducible COX2. COX2 increases under various pathological conditions. Many studies have proven that COX2 participates in various chronic central nervous system injuries. Some studies have found that the COX2 expression was significantly increased in Alzheimer disease (AD), Parkinson’s disease and amyotrophic lateral sclerosis (Teismann et al., 2003; Consilvio et al., 2004). The selective COX2 inhibitor has a significant protective effect against these diseases. Our previous studies also showed that the COX2 was up-regulated in chronic brain injury in rats caused by aluminum overload (Yu et al., 2014). These results suggested that COX2 has a role in the chronic central nervous system injury. However, its effect on T2DM-induced brain injury is still unclear. COX2 exerts its physiological effects by producing PGs. PGD2 is the most abundant prostaglandin in the brain. PGD2 is involved in the regulation of body temperature, the sleep–wake cycle, blood flow, neurotransmission and pain responses (Abdel-Halim et al., 1980; Chiu and Richardson, 1985). It has been shown that PGD2 has both protective and damaging effects in various models of the central nervous system. Therefore, the effect of PGD2 in brain injury remains controversial. However, to our knowledge, the relationship between COX2/PGD2 and T2DM-induced brain injury has never been reported.

Autophagy is a basic biological process which widely exists in normal cells. It plays an important role in maintaining intracellular homeostasis and the health of the body (Lin et al., 2013). Autophagy is involved in the regulation of cell defense and stress, and it will digest and degrade the unfunctional proteins and organelles within the cell (Kirkin et al., 2009). In cells, autophagy contributes to recycling raw materials, updating organelles and maintaining the cellular microenvironment stability (Klionsky, 2008). Studies have demonstrated that autophagy is involved in tumors, infectious diseases, liver diseases, diabetes, and neurodegenerative diseases (Rubinsztein et al., 2012). Some studies showed that the natural process of brain aging also accompanied a chronic and late-onset deterioration of a neuronal autophagy–lysosomal system in AD (Ling and Salvaterra, 2011). Some studies indicated that the decreased expression of LC3BII and the increased expression of p62 were involved in autism-induced hippocampus injuries (Zhang et al., 2016).

The decrease of autophagy level may be an important factor in T2DM-induced organ damage. A significant increase of p62-positive β-cells was found in the autopsy of T2DM patients, indicating that autophagy was inhibited (Mizukami et al., 2014). The autophagy level was decreased in streptozotocin (STZ)-induced type 2 diabetes heart disease (Wang et al., 2014). Han et al. (1992) first reported that the autophagy level in near-loop tubule was decreased in the STZ-induced rat nephropathy model. In addition, some studies have shown that the autophagy level of podocytes was inhibited in STZ-induced diabetic mice and that the autophagy level of high glucose-cultured podocytes also significantly decreased *in vitro* (Fang et al., 2013). Some studies suggested that the autophagy level was significantly decreased in the animal model of T2DM-induced brain injury (Carvalho et al., 2015; Candeias et al., 2018). However, the mechanism of the decrease of autophagy level in T2DM-induced brain injury is still unclear. It is well known that inflammation and apoptosis are the main reasons of organ damage caused by COX2. A recent study has found that high expression of COX2 lowers the expression of LC3BII (Wang L.F. et al., 2015). Celecoxib, a COX2 inhibitor, significantly increased the LC3BII expression and consequently enhanced the autophagy level (Zhu et al., 2017). These results suggest that the decrease of autophagy level is another important reason for organ damage caused by COX2. The PGD2 is the most abundant prostaglandin in the brain. Therefore, we think that COX2–PGD2 may be involved in the mechanism of T2DM-induced brain injury through inhibiting autophagy.

## Materials and Methods

### Animals

Sprague-Dawley (SD) rats were housed in the barrier housing facility, in keeping with the national standard of “Laboratory Animal-Requirements of Environment and Housing Facilities.” The care of the laboratory animal and the animal experimental operation conform to the “Chongqing Administration Rule of Laboratory Animal.” The experimental procedures were approved by the animal laboratory administrative center and the institutional ethics committee of Chongqing Medical University (License number: SYXK YU 2012-0001) and are also in accordance with the National Institutes of Health guidelines. The rats were kept in controlled conditions of temperature (24 ± 2°C), relative humidity (60 ± 10%) and 12/12 h light/dark cycle (light from 08:00 am to 08:00 pm).

To establish the rat model of T2DM (Li et al., 2016; Ma et al., 2017), 60 male rats (80–100 g, 4-week old) were a fed high fat diet (HFD) (20% sugar, 10% lard, 10% egg yolk, and 60% basal feed) after a week of normal diet. After 4 weeks, rats were injected once with low-dose STZ (Solarbio, China) (STZ, 30 mg/kg i.p) to induce partial insulin deficiency, and then continuously fed HFD for 4 weeks after injection of STZ. 30 male rats were alive after the completion of modeling. 30 male rats were randomly and equally divided into the following 3 groups: model group, the low dose meloxicam group (mg⋅kg^-1^), and the high dose meloxicam group (3 mg⋅kg^-1^), *n* = 10 for each group. Then the model rats were orally administrated the COX2 inhibitor (meloxicam) for 8 weeks. There were 9 rats remaining in each group when the administration was completed. The rats of the normal group were fed a normal diet. Before the rats were killed, the rats were weighed, the blood glucose levels were tested using Johnson one touch Ultra Test Strips on Johnson Performa blood glucose meter, and plasma was collected.

### Morris Water Maze Test

Morris water maze was used to evaluate spatial learning and memory function of rat in each group (Kemppainen et al., 2014; Tian et al., 2016). Rats were given four trials per day for four consecutive days. A different entry site was used for each daily session. During each trial, the rats were introduced into the water where a hidden platform was submerged under the water. If rats failed to reach the platform within 90 s, they were gently guided to it and allowed to remain for 10 s on top of the platform. On the 5th day, following the last day of training, rats were introduced into the pool from the entry site where the last training was performed in order to assess retention of the platform location. During this probe trial, the platform was removed from the maze. The latency for the rat to find the hidden platform and the number of times to cross the platform were recorded, with a maximum of 90 s.

### Histopathological Observation

After the Morris water maze test, 3 rats from each group were perfused with heparinized saline (30 ml) to remove blood from the vasculature, and then with 4% paraformaldehyde in phosphate buffered saline (50 ml). The whole brain was then removed and stored in the same fixative. After paraffin embedding, 5-μm sections were obtained and stained with hematoxylin-eosin (H&E). Morphologic changes of hippocampus and cortical neurons were examined using light microscopy. High power fields were sampled from the hippocampus CA1 subfield. Cells with a distinct nucleus and nucleolus were regarded as intact neurons.

### Enzyme-Linked Immunosorbent Assay (ELISA)

Rats cortex and hippocampus from each group of rats (*n* = 6) were removed on the second day after the administration was completed. PGD2 (*Meibiao, Jiangsu, China*) was detected with ELISA kits. Insulin (*Meibiao, Jiangsu, China*) of plasma was detected with ELISA kits (*n* = 9).

### Immunohistochemical Staining Test

Immunohistochemistry was performed to investigate the expression of COX2 in the rat brains. Briefly, brain sections of 3 rats from each group were dewaxed and rehydrated in decreasing concentrations of ethanol. Then the sections were blocked for endogenous peroxidase in 3% H_2_O_2_ in methanol for 20 min at room temperature. Slides were washed with PBS for three times and pre-incubated in 1% serum for 30 min at room temperature. Thereafter, the sections were incubated with primary antibodies COX2 (dilution 1:50, *Santa, United States*) overnight at 4 C. Then, the sections were incubated with a biotinylated secondary antibody (dilution 1:100) for 30 min at 37°C, and incubated with streptavidin for 20 min, and then rinsed for another 3 min × 3 min with PBS before reaction with the DAB solution. The sections were counterstained with hematoxylin and then observed under a microscope.

### Western Blotting Test

Fifty mg of rat cortex and hippocampus (*n* = 4) were added to 0.5 ml of tissue lysate solution for protein extraction and centrifugation at 12,000 × *g* for 10 min at 4°C, and the supernatant was used for the detection of protein concentrations with a BCA protein assay kit *(Beyotime, China)*. A 10 μL sample of protein was separated by sodium dodecyl sulphate polyacrylamide gel electrophoresis (SDS-PAGE) and transferred to PVDF membranes (*Millipore, United States*). The membranes were blocked with 5% BSA for 1 h at room temperature and then probed with specific primary antibodies, including anti-Aβ (1:1000; *Abcam, United Kingdom*), COX2 (dilution 1:400, *Santa, United States*), p-AKT(S473, 1:400, *Santa, United States*), p-AMPK(T172, 1:1000; *Abcam, United Kingdom*), Beclin1(1:500; *Abcam, United Kingdom*), LC3BII (1:1000; *CST, United States*), and p62(1:1000; *CST, United States*) and β-actin (1:4000; *Proteintech, United States*) overnight at 4°C. The membranes were washed three times in TBST and incubated with HRP-conjugated secondary antibodies at room temperature for 1 h. Following four washes in TBST, protein signals were visualized by ECL *(Bio-Rad, United States)*.

### Statistical Analysis

Data are presented as mean ± standard deviation (SD). Statistical analysis was carried out using SPSS statistics software (Version 20.0) and data were analyzed by performing one-way analysis of variance (ANOVA) followed by *post hoc* Tukey’s test. *P*-value less than 0.05 was considered statistically significant.

## Results

### Changes of Body Weight, Blood Glucose and Plasma Insulin in T2DM Rat

**FIGURE 1:**
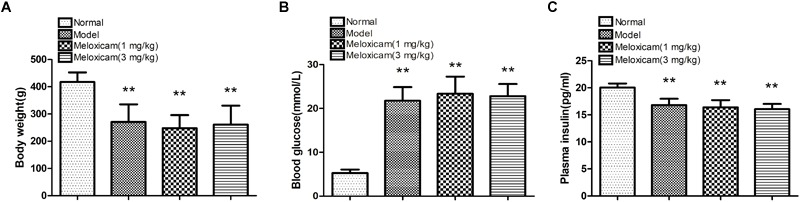
Changes of body weight, blood glucose and plasma insulin in T2DM rat. **(A)** Body weight; **(B)** blood glucose; **(C)** plasma insulin. Rats in the model group showed a significant increase of blood glucose, and a significant decrease of body weight and plasma insulin. The COX2 inhibitor have no effect on the body weight, blood glucose, and plasma insulin. Data are expressed as mean ± SD of nine individual rats in each group, and were analyzed statistically using one-way ANOVA, followed by *post hoc* Tukey’s test. ^∗∗^*P* < 0.01 compared with normal group.

To determine whether our model was a T2DM model, we measured the body weight, blood glucose, and plasma insulin of the rats. Compared with the normal group, model and meloxicam-treated rat’s body weight were significantly decreased. Compared with the normal group, model and meloxicam-treated rats’ blood glucose was significantly increased. Compared with the normal group, the plasma insulin was significantly decreased in model and meloxicam-treated rats. The increase of blood glucose and the decrease of body weight and plasma insulin are consistent with the basic pathological features of T2DM. The COX2 inhibitor has no effect on the body weight, blood glucose, and insulin content of T2DM rats (Figure 1).

### Changes of Spatial Learning and Memory Function in T2DM Rat

**FIGURE 2:**
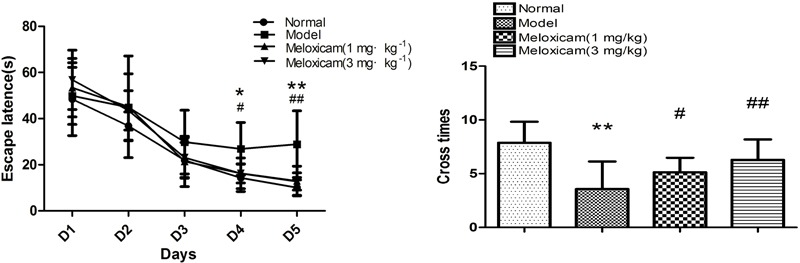
Changes of spatial learning and memory function in T2DM rat. Rats in the model group showed a significant increase of escape latency and a significant decrease of the number of platform cross times. The COX2 inhibitor could significantly blunt those changes caused by T2DM. Data are expressed as mean ± SD of nine individual rats in each group, and were analyzed statistically using one-way ANOVA, followed by *post hoc* Tukey’s test. ^∗^*P* < 0.05 and ^∗∗^*P* < 0.01 compared with normal group; ^#^*P* < 0.05 and ^##^*P* < 0.01 compared with model group, respectively.

**FIGURE 3:**
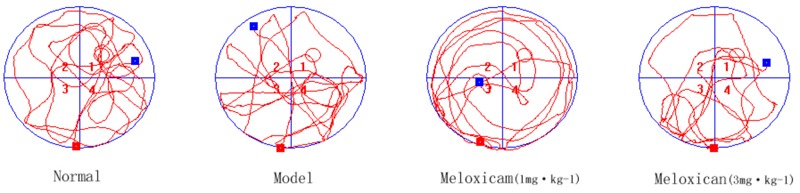
Typical trajectories of the orientation test on day 5 of the water maze test. The circle represents previous platform location in the trace images. The red square represents the starting position of the rat. The blue square represents the end position of the rat.

In order to determine whether rats had neurological impairment, we used the morris water maze to test the learning and memory function of rats. The morris water maze results showed that rats in all groups exhibited a rapid reduction in their escape latencies to find the platform over the 4 training days. Compared with the normal group, rats in the model group showed significantly prolonged escape latency and induced a decrease in the number of platform cross times on 4th and 5th days. Compared with the model group, the escape latency of rats in COX2 inhibitor groups significantly decreased and platform crossing times significantly increased on the 4th and 5th days (Figure 2, 3).

### Changes of Neuronal Pathomorphology in T2DM Rat

**FIGURE 4:**
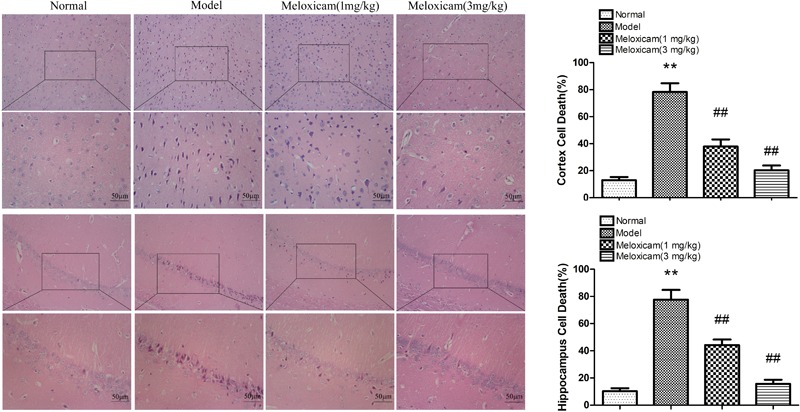
Changes of neuronal pathomorphology in T2DM rat cortex and hippocampus (HE × 200, ×400, Scale bar = 50 μm). The hippocampus and cortical neurons were in distinct and regular structure, and arranged densely and clearly in the normal group. Compared to normal group, neuron in the model group showed a remarkable karyopyknosis. The COX2 inhibitor significantly improved karyopycnosis. Data are expressed as the mean ± SD for three individual rats in each group, and were analyzed statistically using one-way ANOVA, followed by *post hoc* Tukey’s test. ^∗∗^*P* < 0.01 compared with normal group; ^##^*P* < 0.01 compared with model group, respectively.

In order to determine whether the rat’s brain had pathological changes, we used HE to test the changes of neuronal pathomorphology in rats’ hippocampus and cortex. In the control group, the morphological neuronal structure of the hippocampus and cortex was intact and clear. By comparison, the neuron in the model group showed a remarkable karyopyknosis. COX2 inhibitor significantly improved neuronal karyopycnosis (Figure 4).

### Changes of COX2 Expression in T2DM Rat Hippocampus and Cortex

**FIGURE 5:**
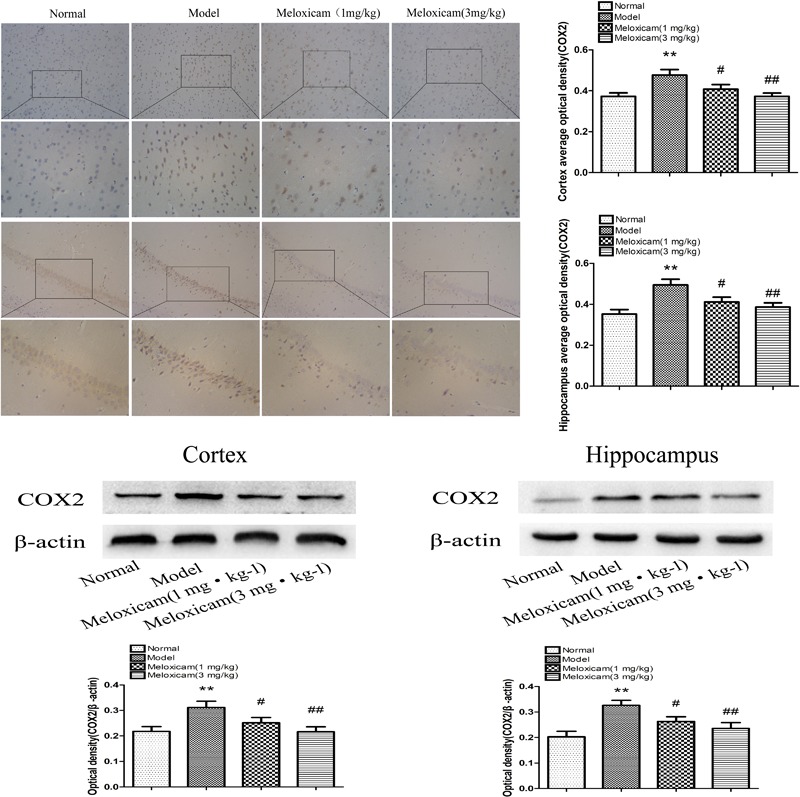
Changes of COX2 expression in T2DM rat cortex and hippocampus (×200, ×400). Compared with normal group, the COX2 expression in cortex and hippocampus were significantly increased compared with normal group. The COX2 inhibitor significantly reversed the increased of cortex and hippocampus COX2 expression. Data are expressed as the mean ± SD for three (IHC) and four (WB) individual rats in each group, and were analyzed statistically using one-way ANOVA, followed by *post hoc* Tukey’s test. ^∗∗^*P* < 0.01 compared with normal group; ^#^*P* < 0.05 and ^##^*P* < 0.01 compared with model group, respectively.

COX2 is a rate-limiting enzyme in the synthesis of PGs. In order to determine whether COX2-PGD2 was involved in brain damage in T2DM, we used IHC and WB to test COX2 expression in the rat’s cortex and hippocampus. The expression of COX2 in the model rat’s cortex and hippocampus were significantly increased compared with the control group. COX2 inhibitor significantly blunted the increase of COX2 protein expression in the model rat’s cortex and hippocampus (Figure 5).

### Changes of PGD2 Content in T2DM Rat Hippocampus and Cortex

**FIGURE 6:**
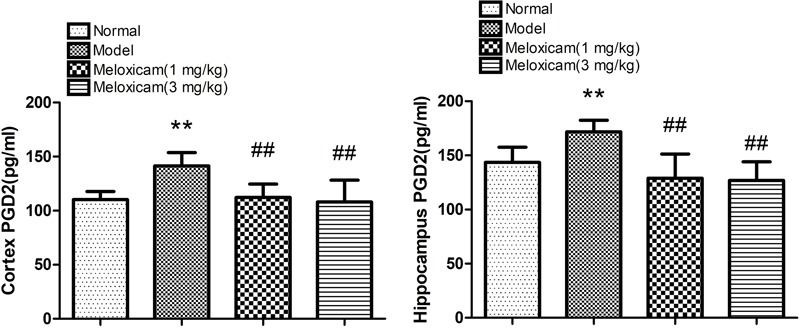
Effect of COX2 inhibitor on changes of hippocampus and cortex PGD2 content in T2DM rat cortex and hippocampus. Compared with that of normal group, the contents of PGD2 significantly increased in model group. Compared with that of model group, the administration of COX2 inhibitor significantly decreased the content of PGD2. Data are expressed as the mean ± SD for six individual rats in each group, and were analyzed statistically using one-way ANOVA, followed by *post hoc* Tukey’s test. ^∗∗^*P* < 0.01 compared with normal group. ^##^*P* < 0.01 compared with model group, respectively.

In order to determine whether COX2-PGD2 was involved in brain damage in T2DM, we used Elisa to test PGD2 content in the rat’s cortex and hippocampus. PGD2 content of the rat’s hippocampus and cortex in the model group were significantly increased compared with the control group. The administration of COX2 inhibitor significantly blunted the increase of PGD2 content in diabetes rats (Figure 6).

### Changes of Aβ, p-AKT(S473), p-AMPK(T172), Beclin1, LC3BII and p62 Expressions in T2DM Rat Hippocampus and Cortex

**FIGURE 7:**
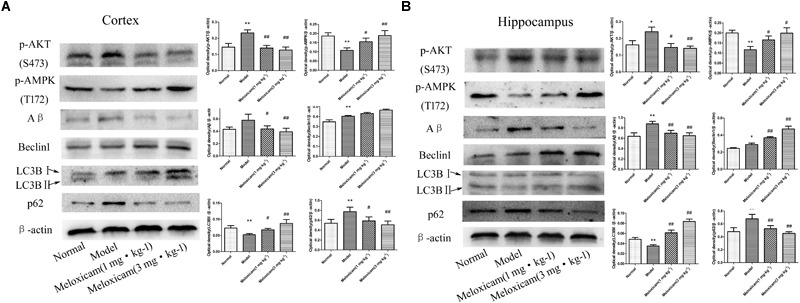
Changes of p-AKT(S473), p-AMPK(T172), Aβ, Beclin1, LC3BII, and p62 expressions in rat cortex and hippocampus. **(A)** The changes of proteins in cortex. **(B)** The changes of proteins in hippocampus. Compared with normal group, the expressions of p-AKT(S473), Aβ, Beclin1, and p62 were significantly increased, while p-AMPK(T172) and LC3BII expression was significantly decreased in the model group. The COX2 inhibitor significantly increased the expression of p-AMPK(T172), Beclin1 and LC3BII, while significantly decreased the expression of p-AKT(S473), Aβ, and p62. The relative protein level was standardized to endogenous β-actin protein for each sample. Data are expressed as mean ± SD of four individual rats in each group, and were analyzed statistically using one-way ANOVA, followed by *post hoc* Tukey’s test. ^∗^*P* < 0.05 and ^∗∗^*P* < 0.01 compared with normal group; ^#^*P* < 0.05 and ^##^*P* < 0.01 compared with model group, respectively.

To determine whether COX2-PGD2 induced T2DM brain injury by affecting p-AKT(S473) and p-AMPK(T172) to inhibit autophagy and reduce the clearance of Aβ, we used WB to detect the related protein expression in the rat’s cortex and hippocampus. Compared with the control group, the expressions of p-AKT(S473), Aβ, Beclin1, and p62 were significantly increased, while LC3BII and p-AMPK(T172) expression was significantly decreased in the model rat’s cortex and hippocampus. The COX2 inhibitor significantly increased the expression of p-AMPK(T172), Beclin1 and LC3BII, and significantly decreased the expression of p-AKT(S473), Aβ and p62 (Figure 7).

## Discussion

The increase in the prevalence of T2DM has become an important threat for people’s health. T2DM-induced brain injury has been a focus in many studies (Sebastião et al., 2014). Many studies have proven that COX2 is involved in various chronic central nervous system injuries, and the mechanism of the damage is related to inflammation and apoptosis. Autophagy is decreased and inversely correlated with COX2 expression in nasal polyps (Wang L.F. et al., 2015). Celecoxib, a COX2 inhibitor, could significantly increase the LC3BII expression to enhance autophagy level in human prostate cancer PC3 cells (Zhu et al., 2017). These results suggested that autophagy inhibition is an injury mechanism of COX2. In our study, we found the functions of learning and memory to be significantly impaired, and the cortex and hippocampus neurons had significant nuclear pyknosis in the model rats. The expressions of COX2, Beclin1 and p62 were significantly increased and that of LC3BII was significantly decreased in the cortex and hippocampus of model rats. The PGD2 content was significantly increased in the cortex and hippocampus of model rats. Beclin1 is a key protein in the initial stage of autophagy and promotes the formation of the autophagosome (Reggiori et al., 2005; Mizushima, 2010). However, this autophagosome is still not fully functional. LC3BII represents the effect of strength of the autophagosome, and the degradation of p62 expression by the autophagosome reflects the level of autophagic flux (Klionsky et al., 2010). In the cortex and hippocampus of model rats, we found that the expression of Beclin1 was significantly increased. However, the expression of LC3BII was decreased, thus indicating the lower effect strength of the autophagosome to decrease the degradation of p62, which consequently allowed for significantly increased expression of p62. Therefore, the autophagy level was decreased in the cortex and hippocampus of model rats. Our results suggested that the increase of COX2–PGD2 levels caused the decrease of autophagy in T2DM-induced brain injury.

In agreement with our results, studies showed that platelet-rich plasma induced chondroprotection, which was associated decreased COX2 and increased the autophagy level in human osteoarthritic cartilage (Moussa et al., 2017). Morin hydrate can decrease the COX2 expression and increase the autophagy level in the treatment of atherosclerosis (Zhou et al., 2017). Tart cherries can significantly improve the working memory of normal aged rats; this was accompanied with significantly decreased COX2 expression and significantly increased autophagy level in the hippocampus (Thangthaeng et al., 2016). Some researchers found that the inhibition of autophagic flux induced M1 microglial phenotype with a higher level of COX2 (Xia et al., 2016) and that M1 microglial phenotype is deleterious (Huang and Feng, 2013). Taken together, these results suggested that the decrease of autophagy may be an important causative factor of tissue injury caused by COX2 overexpression.

COX2-mediated autophagy inhibition may be due to the increase of PG production. Our previous studies found significantly increased COX2 and PGD2 levels in aluminum-overloaded rat brains (Wang H. et al., 2015; Ma et al., 2016). PGD2 may be related to the development and migration of microglia cells, which may be involved in various nerve injuries (Mohri et al., 2003). DP1 and DP2 are the corresponding receptors for PGD2. DP1 can stimulate adenylate cyclase to increase the intracellular cyclic adenosine monophosphate (cAMP) content and then activate PKA. DP2 can stimulate the PI3K/AKT/mTOR pathway (Hata and Breyer, 2004). Some studies indicated that PGD2 could increase the expression of p-AKT(S473) (Wang et al., 2008). Those results suggested that PGD2 increases the expression of AKT through DP2. Although it is known that PKA is involved in the regulation of autophagy (Torres-Quiroz et al., 2015), its exact role in autophagy remains unclear. The PI3K/AKT/mTOR pathway is a classical regulatory pathway of autophagy, and the activation of the PI3K/AKT/mTOR pathway can inhibit the expression of Beclin1 and LC3BII to decrease the autophagy level (Meijer and Codogno, 2006). Our results showed that p-AKT(S473), Beclin1 and p62 expressions were significantly increased, whereas the p-AMPK(T172) and LC3BII expression was significantly decreased in the cortex and hippocampus of model rats. Therefore, Our results suggest that PGD2 is involved in the mechanism of autophagy inhibition.

AMPK is a key protein in the regulation of autophagy. The activation of AMPK can decrease mTOR expression to promote autophagy. Kainuma reported that p-AMPK(T172) is induced by PGD2 in MC3T3-E1 cells (Kainuma et al., 2015). However, in our study, the expression of p-AMPK(T172) was significantly decreased in the cortex and hippocampus of T2DM rats. The reason for the difference in p-AMPK(T172) expression between our’s and Kainuma’s results may be explained as follows: (1) The tissue material for the detection of p-AMPK(T172) expression is different. Osteoblastic cell lines (MC3T3-E1) were used in Kainuma’s study, while rats cortex and hippocampus were used in our study. However, whether this difference in the material used for detection can affect the experimental results is not clear. (2) The stimulating pattern for p-AMPK(T172) expression is not exactly the same. MC3T3-E1 cells were only treated with PGD2 in Kainuma’s study. In contrast, in our study, rats were treated with a high-fat diet (HFD) and STZ, which further increased the PGD2 and blood glucose. (3) The cell/rat processing times are different (reflecting the difference between an acute and chronic disease model). The MC3T3-E1 cells were only treated with PGD2 for 1 min to 60 min in Kainuma’s study. In contrast, in our study, rats were treated with a HFD for 8 weeks and STZ once during week 4, consistent with a chronic injury model.

Some experimental findings are similar to ours. The effects of PGD2 were different in different models; for example, a decrease of PGD2 was associated with depressive behavior (Chu et al., 2017), and PGD2 promoted apoptosis in transgenic APP/PS1 mice (Guo J.W. et al., 2017). Moreover, the expression of p-AMPK(T172) changes with time in focal cerebral ischemia injury (Fu et al., 2016).

Similar to our results, the expression of p-AMPK(T172) was significantly decreased in the cortex of transgenic db/db DM mice and STZ-induced DM rats (Liu et al., 2016; Peng et al., 2016). The reason for the decrease in p-AMPK(T172) may be related to p-AKT(S473). Moreover, the expression of p-AKT(S473) was significantly increased in the cortex of db/db mice (Peng et al., 2016). We also found that the expression of p-AKT(S473) was significantly increased in the cortex and hippocampus in the rat model. Some studies have demonstrated that p-AKT(S473) can significantly reduce the expression of p-AMPK(T172) by inhibiting liver kinase B1(LKB1) (Kovacic et al., 2003; Soltys et al., 2006). LKB1 can be expressed in the brain, where it has important effects (Ryan et al., 2017).

The massive generation and deposition of Aβ is a major pathological feature in AD brain. It is known that diabetes can significantly increase the risk of AD (Ott et al., 1999). The Aβ expression in rat brain capillaries increased 2.5 times after oxygen and glucose deprivation (Bulbarelli et al., 2012), and the expression of Aβ in the hippocampus also increased significantly in scopolamine-induced memory impairment (Hafez et al., 2017). Taken together, these studies suggested Aβ as a direct reason of central nervous system injury. Our experimental results also showed that the expression of Aβ was significantly increased in T2DM rat brains and that meloxicam significantly decreased the Aβ expression. Similar with our results, it was reported that Aβ level was significantly increased in the brain of T2DM patients (Yang and Song, 2013), and the levels of Aβ and its precursor were significantly increased in the forebrain cortex of T2DM rats (Li et al., 2007). Early studies suggested that fibrous Aβ is neurotoxic and is responsible for the death of nerve cells. However, recent studies have shown that soluble Aβ is an important source of neurotoxicity and is more toxic than fibrous Aβ (Hernandez et al., 2010). Some studies have also shown that soluble Aβ, which is isolated from the brain tissue of AD patients, can induce hyperphosphorylation of tau protein in hippocampal neurons and damage the cytoskeleton of microtubules (Jin et al., 2011).

The inhibition of COX2 may be an effective way to enhance autophagy level by decreasing the PGD2 content, and then, the Aβ will get cleared. In meloxicam-treated rats, the cognitive dysfunction was significantly improved, and the content of PGD2 was significantly decreased in the cortex and hippocampus. Furthermore, the expressions of COX2, p-AKT(S473), Aβ and p62 were significantly decreased, and the expression of p-AMPK(T172), Beclin1 and LC3BII was significantly increased in the cortex and hippocampus. The COX2 inhibitor decreased the PGD2 content to alleviate the activation of DP2. The decreased activation of DP2 is expected to decrease the expression of the PI3K/AKT/mTOR pathway, and then, the increased expression of Beclin1 and LC3BII may improve the levels of autophagy. However, the effect of DP1 (PKA pathway) on autophagy remains unknown. Some studies indicated that the PKA inhibitor significantly increased the LC3BII expression in the rats hippocampus (Azimi et al., 2016), and another study indicated that neuropeptide Y via PKA stimulates autophagy in hypothalamic neurons to delay aging and produce protective effects against hypothalamic impairments associated with age (Aveleira et al., 2015). The decreased expression of p-AKT(S473) will increase the expression of p-AMPK(T172), and then, the increase expression of Beclin1 and LC3BII may promote autophagy. The effect of DP1 (PKA pathway) on autophagy is still controversial and needs further verification in T2DM-induced brain injury by another experiments. However, our results indicated that the ultimate result of the decrease of PGD2 was increased autophagy levels in T2DM-induced brain injury.

In our studies, we found that Beclin1 expression was not coherent with the behavior of LC3II and p62. Our results found that PGD2 was significantly increased in the cortex and hippocampus of model rats. In many studies, Beclin1 was reported to be increased not only in chronic injuries, such as T2DM-induced brain injury (Carvalho et al., 2015), but also acute injuries, such as cerebral ischemia stroke (Guo D. et al., 2017). These results suggested that Beclin1 may show a compensatory increase in the sustained protection of the cortex and hippocampus of model rats. The decrease in the expression of LC3BII represents the lower efficacy of the autophagosome to decrease the degradation of p62; consequently, the expression of p62 was then significantly increased. Therefore, the expression of Beclin1 and p62 was significantly increased and the expression of LC3BII was significantly decreased in the cortex and hippocampus of model rats compared with the normal rats. The autophagy level was significantly decreased in the cortex and hippocampus of model rats. The expression of Beclin1 and LC3BII was significantly increased when the COX2 inhibitor treatment was performed to decrease the expression of mTOR by decreasing the PGD2 content to decrease the expression of p-AKT(S473) and increase the expression of p-AMPK(T172). The number and effect strength of the autophagosome significantly increased, and then, the expression of p62 significantly decreased. Therefore, compared with the model rats, the expression of Beclin1 and LC3BII was significantly increased and that of p62 was significantly decreased when the COX2 inhibitor treatment was performed in the cortex and hippocampus of meloxicam-treated rats. The autophagy level was increased in the cortex and hippocampus of meloxicam-treated rats. Conversely, recent studies found that p62 stabilizes COX2 protein through the p62 ubiquitin-associated domain (Sample et al., 2017). The increase of p62 can aggravate the COX2/PGD2-induced damage. The inhibition of COX2 not only decreases the expression of COX2 but also reduces the level of p62 by increasing the autophagy levels.

In summary, our present experimental results suggested that the activation of COX2-PGD2 pathway is involved in the mechanism of brain injury caused by T2DM through inhibiting autophagy to decrease the Aβ clearance. These findings pointed out that the COX2-PGD2 pathway is a potential therapeutic target for T2DM brain injury.

## Author Contributions

JY made substantial contribution to conception, design, and performance of the study. YY, QC, QZ, YLu, YX, WD, HW, HL, LY, CH, JZ, YLi, HX, ZC, JM, and XT participated in performance of all experiments and carried out the data analysis. YY participated in performance of the study and in writing the manuscript. All authors read and approved the final manuscript.

## Conflict of Interest Statement

The authors declare that the research was conducted in the absence of any commercial or financial relationships that could be construed as a potential conflict of interest.
